# Single-Cell Co-expression Analysis Reveals Distinct Functional Modules, Co-regulation Mechanisms and Clinical Outcomes

**DOI:** 10.1371/journal.pcbi.1004892

**Published:** 2016-04-21

**Authors:** Jie Wang, Shuli Xia, Brian Arand, Heng Zhu, Raghu Machiraju, Kun Huang, Hongkai Ji, Jiang Qian

**Affiliations:** 1 The Wilmer Eye Institute, Johns Hopkins University School of Medicine, Baltimore, Maryland, United States of America; 2 Department of Neurology, Johns Hopkins University School of Medicine, Baltimore, Maryland, United States of America; 3 Hugo W Moser Research Institute at Kennedy Krieger, Johns Hopkins University School of Medicine, Baltimore, Maryland, United States of America; 4 Department of Computer Science and Engineering, the Ohio State University, Columbus, Ohio, United States of America; 5 The Sidney Kimmel Comprehensive Cancer Center, Johns Hopkins University School of Medicine, Baltimore, Maryland, United States of America; 6 Department of Pharmacology and Molecular Sciences, Johns Hopkins University School of Medicine, Baltimore, Maryland, United States of America; 7 Center for High-Throughput Biology, Johns Hopkins University School of Medicine, Baltimore, Maryland, United States of America; 8 Department of Biomedical Informatics, the Ohio State University, Columbus, Ohio, United States of America; 9 Department of Biostatistics, Johns Hopkins University Bloomberg School of Public Health, Baltimore, Maryland, United States of America; University of Southern California, UNITED STATES

## Abstract

Co-expression analysis has been employed to predict gene function, identify functional modules, and determine tumor subtypes. Previous co-expression analysis was mainly conducted at bulk tissue level. It is unclear whether co-expression analysis at the single-cell level will provide novel insights into transcriptional regulation. Here we developed a computational approach to compare glioblastoma expression profiles at the single-cell level with those obtained from bulk tumors. We found that the co-expressed genes observed in single cells and bulk tumors have little overlap and show distinct characteristics. The co-expressed genes identified in bulk tumors tend to have similar biological functions, and are enriched for intrachromosomal interactions with synchronized promoter activity. In contrast, single-cell co-expressed genes are enriched for known protein-protein interactions, and are regulated through interchromosomal interactions. Moreover, gene members of some protein complexes are co-expressed only at the bulk level, while those of other complexes are co-expressed at both single-cell and bulk levels. Finally, we identified a set of co-expressed genes that can predict the survival of glioblastoma patients. Our study highlights that comparative analyses of single-cell and bulk gene expression profiles enable us to identify functional modules that are regulated at different levels and hold great translational potential.

## Author Summary

With the development of single-cell sequencing, an increasing number of biological insights were revealed at the single-cell resolution. Here we integrated the expression profiles from single cells and bulk tissues to discover that a majority of gene pairs were specifically co-expressed at single-cell and bulk levels. Our comparative analysis reveals co-expressed functional modules at different levels, and suggests a distinct regulatory mechanism in which single-cell co-expressed genes are regulated through physical interactions from different chromosomes. Moreover, we found a set of co-expressed genes to predict patient survival. This study suggests that single-cell and bulk co-expression analysis could provide novel biological insights and great clinical potential.

## Introduction

Gene expression is often coordinated to carry out cellular activities and biological functions [1]. If the expression levels of two genes rise and fall together across different conditions, they are likely to be members of the same protein complex or participate in the same biological pathways. Therefore, co-expression analysis has been widely used to predict protein-protein interactions (PPIs) or annotate functions of uncharacterized genes [2–4]. Built upon co-expression relationships, co-expression networks were often constructed to reveal the functional modules consisting of genes with functional relationships [5–7]. Furthermore, co-expression relationships are often considered to be the consequence of co-regulation that is governed by the same regulatory machinery. Therefore, regulatory elements could be predicted based on the co-expression relationships [8–10]. In addition, co-expression analysis has been applied to cancer biology. For example, co-expressed gene sets could reveal interaction modules in tumor progression [11], or serve as molecular signatures to classify tumors into different subtypes, which often showed distinct clinical outcomes [12,13].

Previous co-expression analyses were mainly conducted at the bulk level in which a large population of cells was profiled as a whole. Recently, single-cell sequencing has emerged as a powerful tool to investigate cellular variability and intratumor heterogeneity [14–16]. However, it remains elusive whether co-expression analysis at the single-cell level will provide novel biological insights into the molecular principles of transcription regulation that would be otherwise hidden at the bulk level. For example, can the same set of co-expressed genes be identified both at the single-cell and bulk levels from the same tissue origin? Will the comparative co-expression analysis reveal functional modules that are regulated at different levels? Do the co-expression relationships detected at the single-cell and bulk levels reflect the same regulatory mechanisms?

To address these important questions, we developed a computational approach to perform comparative co-expression analysis between single-cell and bulk samples, and discovered that the majority of the co-expressed gene pairs were unique. Multiple lines of evidence suggest that the discrepancy between the two analyses is not due to technical artifacts. Interestingly, the co-expressed genes in bulk tissues tend to have the same biological functions, while the co-expressed genes in single cells encode proteins that are likely to interact with each other. Strikingly, members in different protein complexes are often predominately connected by one type of co-expression relationships. Furthermore, we find that the co-expression relationships in the single cells and bulk tissues might reflect distinct co-regulatory mechanisms. Interestingly, interchromosomal interactions are highly enriched for single-cell co-expression. Finally, we discover a set of co-expressed genes that can predict the clinical outcome of glioblastoma.

## Results

### Distinct sets of co-expressed genes were identified at the single-cell level

We used glioblastoma as a model system because both single-cell and bulk expression data are available. A dataset of single-cell RNA-seq was obtained from 430 individual cells of five glioblastoma patients [14]. Similarly, gene expression profiles of 120 glioblastomas as bulk tissues were obtained from TCGA consortium [17]. To compare co-expression patterns at single-cell and bulk levels, we calculated Pearson’s correlation coefficients (R) of gene expression for all possible gene pairs across the cells (or tumors).

**Fig 1:**
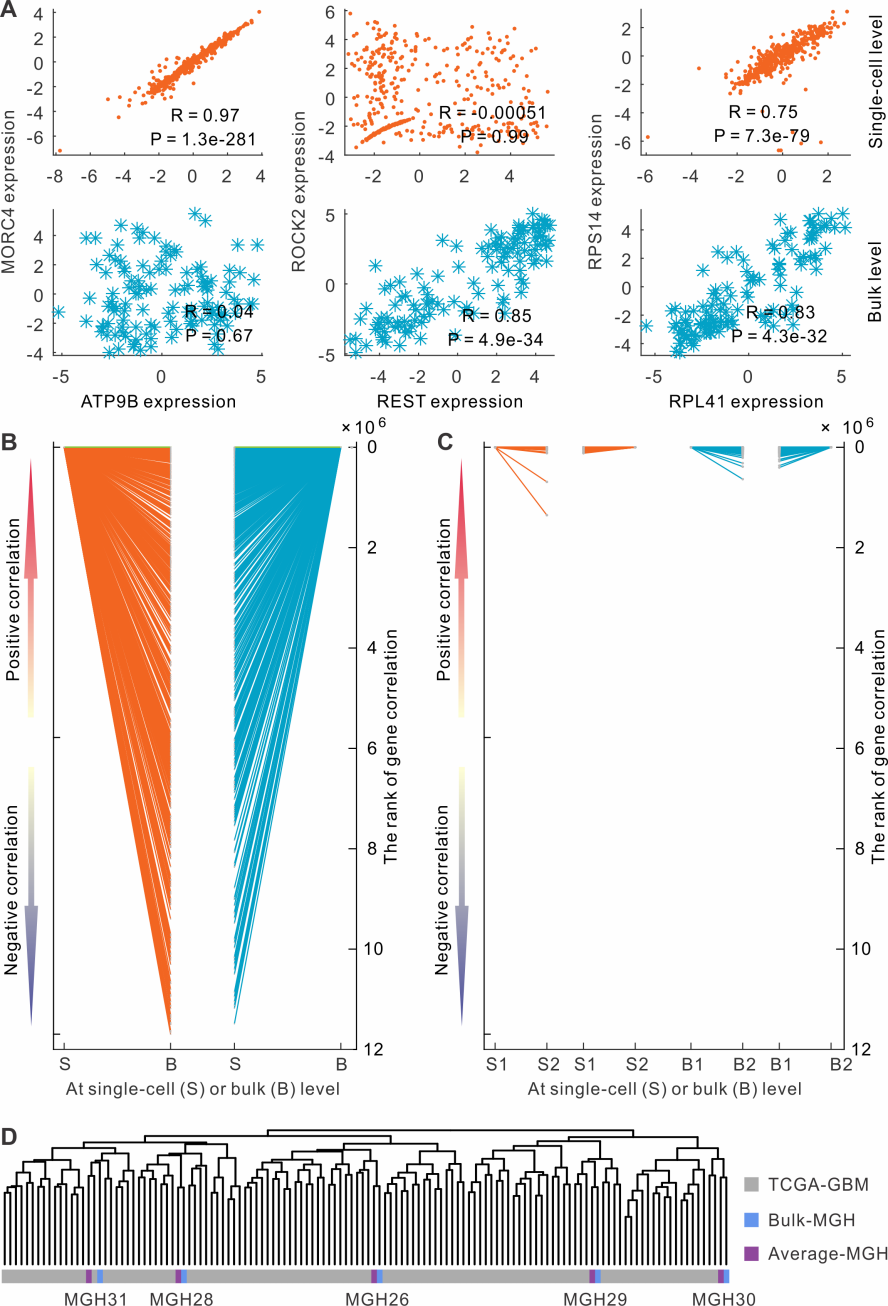
Distinct sets of co-expressed genes were identified for single cells and bulk tissues. (A) Examples of single-cell specific, bulk specific and shared co-expressed gene pairs. (B) The mapping of the top 1,000 positively correlated genes in single cells (or bulk tissues) to their correlation in bulk tissues (or single cells). Each line represent a gene pair. The cells (or tissues) were randomly partitioned into two sub-groups. (C) The mapping of the top 1,000 positively correlated genes in one sub-group to the other sub-group. (D) Clustering of glioblastomas based on gene expression profiles. Bulk samples from TCGA (TCGA-GBM), single-cell-derived average gene expression (Average-MGH) and 5 genuine bulk samples (Bulk-MGH) for single-cell sequencing.

Strikingly, the majority (> 90%) of co-expressed gene pairs were unique to either single-cell or bulk analysis. For instance, we observed that the expression profiles of two genes, *ATP9B* and *MORC4*, were highly correlated at the single-cell level (R = 0.97, Fig 1A); the correlation coefficients calculated separately from the five tumors were also consistent (S1 Fig). However, their correlation was not significant at the bulk level (R = 0.04). Conversely, the expression profiles of *REST* and *ROCK2* were found highly correlated at the bulk level (R = 0.85), but not at the single-cell level (R = 0.00051, Fig 1A and S2 Fig). Globally, we separately identified the top 1,000 most correlated gene pairs at either single-cell or bulk level and cross-examined whether the same pairs were also correlated at the other level. Surprisingly, only 76 (7.6%) of the top 1,000 gene pairs are shared between the bulk and single-cell levels (Fig 1B). For example, *RPL41* and *RPS14* are co-expressed in both single cells (R = 0.75) and bulk tissues (R = 0.83) (Fig 1A and S3 Fig). However, most co-expressed gene pairs at the single-cell level have no or even negative correlation at the bulk level. Similar pattern was also observed for the top 1,000 correlations at the bulk level. It is worthy to note that the observation is not sensitive to the correlation measurement we choose. For example, if maximal information coefficient (MIC), which is able to capture non-linear relationships [18], was used, a consistent pattern was observed that 96.4% co-expressed gene pairs were specific at single-cell or bulk level (S4 Fig). These results suggested that distinct sets of co-expressed gene pairs were yielded at single-cell and bulk levels.

### Distinct co-expression patterns are not due to artifacts

Several lines of evidence suggest that the discrepancy in co-expression analysis between bulk and single-cell levels is not due to technical artifacts. First, we checked whether expression correlation was sensitive to the samples chosen for analysis. We randomly partitioned the cells (or tumors) into two equal-sized sub-groups and separately calculated corresponding gene expression correlations. The top 1,000 co-expressed genes were highly consistent between the two sub-groups (Fig 1C). For example, 524 (52.4%) of the top 1,000 correlations were shared between the two sub-groups in the single-cell analysis. The remaining 47.6% of gene pairs are also highly correlated, even though they were not in the top 1,000. A similar observation was made for bulk-level analysis. This observation suggested that expression correlations were robust and not sensitive to the samples used for calculation.

Second, we examined whether the dissociation and processing of single cells introduced measurement errors, which could lead to the discrepancy of co-expression between single-cell and bulk levels. For the five glioblastomas with single-cell expression profiles, we averaged gene expression across the individual cells and then compared the average gene expression profiles with the genuine bulk expression profiles from the same glioblastomas. The comparison showed that the average gene expression was highly correlated with the expression in bulk tissue for each tumor (Fig 1D and S5 Fig). These results suggest that the procedure of isolating and harvesting single cells did not introduce much distortion in expression profiles. Furthermore, in comparison of the expression profiles of the five tumors for single-cell sequencing with the other 120 bulk tumors from TCGA, we found that the five samples were dispersed among the 120 glioblastomas (Fig 1D). This result suggests that the five tumors for single-cell analysis are not characteristically different to the 120 glioblastomas for bulk analysis, and both of the datasets were representative of primary glioblastomas.

Third, we explored whether the discrepancy of co-expression patterns between single cells and bulk tissues could be observed in other tissues. Similar analyses were performed using data obtained from prostate cancers. We compared the transcriptome of 122 individual prostate cancer cells with those of 398 bulk prostate cancers from TCGA [19]. The results showed that only 4% of the top 1,000 correlations were shared between single-cell and bulk levels (S6 Fig). Taken together, all of the above analyses suggest that the observation of distinct co-expressed gene pairs in single cells and bulk tissues was valid, and not due to technical artifacts.

### Members of protein complexes co-expressed at different levels

In order to dissect the biological roles of the co-expressed genes at the single-cell level, we classified the co-expressed genes into three groups: single-cell specific, bulk specific, and shared at both levels (S7 Fig and see Materials and Methods for the details). In brief, we compared the distributions of expression correlation coefficients from real and randomly shuffled expression profiles to identify the thresholds of significantly positive correlations at single-cell or bulk levels. Using the obtained thresholds, 5,303, 107,851, and 12,584 gene pairs were classified as single-cell specific, bulk specific, and shared co-expressed gene pairs, respectively (S8 Fig).

**Fig 2:**
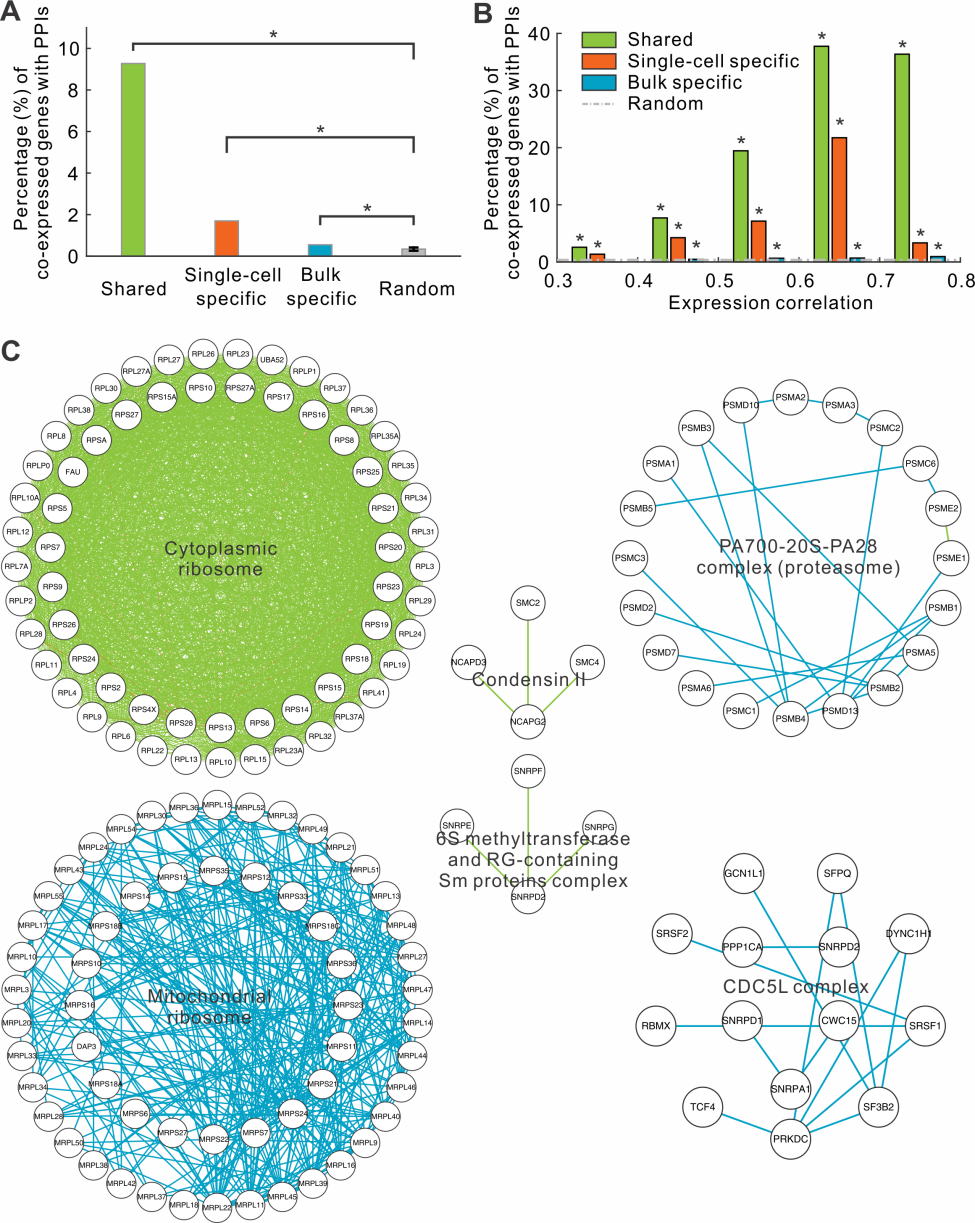
Members in protein complexes are predominately connected by one type of co-expressions. (A) The fraction of co-expressed genes whose protein products interact with each other. (B) The same fraction in function of correlation coefficients. (C) Examples of protein complexes. Two gene members in a complex were connected if they are co-expressed. The color denotes the types of co-expressions: single-cell specific (orange), bulk specific (cyan), and shared (green).

Next, we attempted to discover distinct characteristics of these three groups of co-expressed genes. We first checked whether protein products of the co-expressed genes were enriched for known PPIs. By surveying the PPI networks of the BioGRID database [20] using the corresponding proteins of those co-expressed genes, we found that bulk specific co-expressed genes were slightly enriched for PPIs. Specifically, the protein products of 591 (0.55%) of the 107,851 co-expressed gene pairs specific to the bulk tissues have known PPI relationships, while only 0.34% was expected for randomized gene pairs (P = 3.8E-91, student’s t-test). In contrast, PPIs were much more enriched in single-cell specific co-expressed genes (90 of 5,303, 1.7%), which was a 5-fold enrichment compared to the expectation (P = 2.0E-247, student’s t-test) (Fig 2A). Strikingly, we observed that 1,167 of 12,584 (9.3%) shared co-expressed genes have PPIs (Fig 2A), a 27-fold enrichment compared to the expectation (P < 1.0E-500, student’s t-test). The enrichment was not due to relatively high correlation coefficients in the shared group. The same trend was also observed if we compared the three groups at the same range of correlation coefficients (Fig 2B). Furthermore, the enrichment for PPIs in co-expressed genes increased with the degree of correlation coefficients, suggesting the fidelity of the relationships between the co-expressed genes and PPIs.

Surprisingly, we observed that the three classes of co-expressions were not homogeneously distributed among annotated protein complexes. Instead, different protein complexes were enriched in different classes of co-expressions. Members of many protein complexes are co-expressed at bulk level, such as proteasome and CDC5L complex (Fig 2C). However, members in other complexes (e.g. condensing II) are co-expressed in both single cells and bulk tissues (Fig 2C). Perhaps the most striking examples are cytoplasmic and mitochondrial ribosomes. Of 1,816 co-expressed gene pairs that belong to the cytoplasmic ribosomal complexes, 1,791 (98.6%) were co-expressed at both single-cell and bulk levels. In contrast, among 329 co-expressed genes of the mitochondrial ribosomal complexes, all of them are bulk specific (Fig 2C). These results suggest that the synchronized expression of members in protein complexes is governed through different types of co-expression relationships, reflecting distinct regulatory mechanisms.

### Single-cell specific co-expressed genes are enriched for distinct biological functions

**Fig 3:**
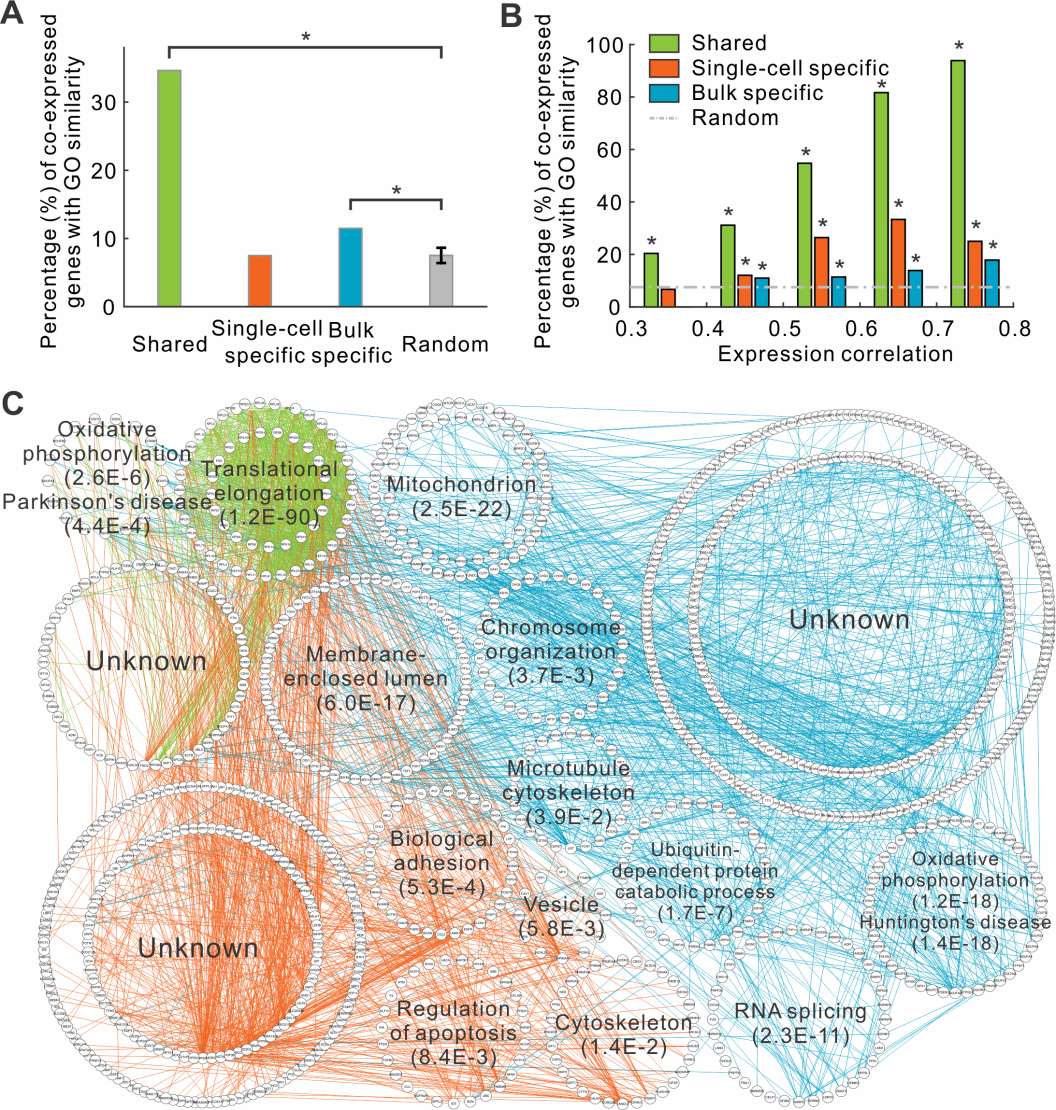
Different types of co-expressions are associated with distinct biological functions. (A) The fraction of co-expressed genes that have the similar biological functions. (B) The same fraction in function of correlation coefficients. (C) Gene function network of top 1,000 co-expressions. Genes with the same functions are placed around circles. Two genes are connected in lines if they have single-cell specific (orange), bulk specific (cyan), or shared (green) co-expression.

We next examined whether co-expressed genes tend to share similar biological functions. To this end, we calculated the semantic similarity of the biological process (BP) terms of gene ontology (GO) [21] between two genes using GOSemSim [22]. Our analyses demonstrated that the shared and bulk specific co-expressed gene pairs tend to have similar biological functions. Specifically, the fractions of shared and bulk specific co-expressed genes having the same functions were 4.6 and 1.5-fold higher than the expectation, respectively (Fig 3A). In contrast, the single-cell specific co-expressed genes were not enriched for function similarity (0.997-fold, Fig 3A). Nevertheless, gene pairs with the highest correlation coefficients (R > 0.4) at the single-cell level were also enriched for function similarity (Fig 3B).

The three groups of co-expressed gene pairs are enriched for different biological functions. Again, we checked the biological functions associated with the genes from the top 1,000 shared, single-cell specific, or bulk specific co-expression pairs. For single-cell specific co-expressed genes, GO terms including biological adhesion, and regulation of apoptosis were enriched (Fig 3C and S9 Fig). The shared co-expressed genes were associated with translational elongation, and oxidative phosphorylation. It is also interesting to note that these genes are also enriched in neurodegenerative diseases, such as Parkinson’s disease, given the neuronal origin of glioblastomas (Fig 3C and S9 Fig). The bulk specific co-expressed genes were significantly associated with oxidative phosphorylation and neurodegenerative diseases. These results further demonstrated that the different functional modules were associated with different types of co-expressed genes.

### Distinct regulatory mechanisms are responsible for three types of co-expressed genes

**Fig 4:**
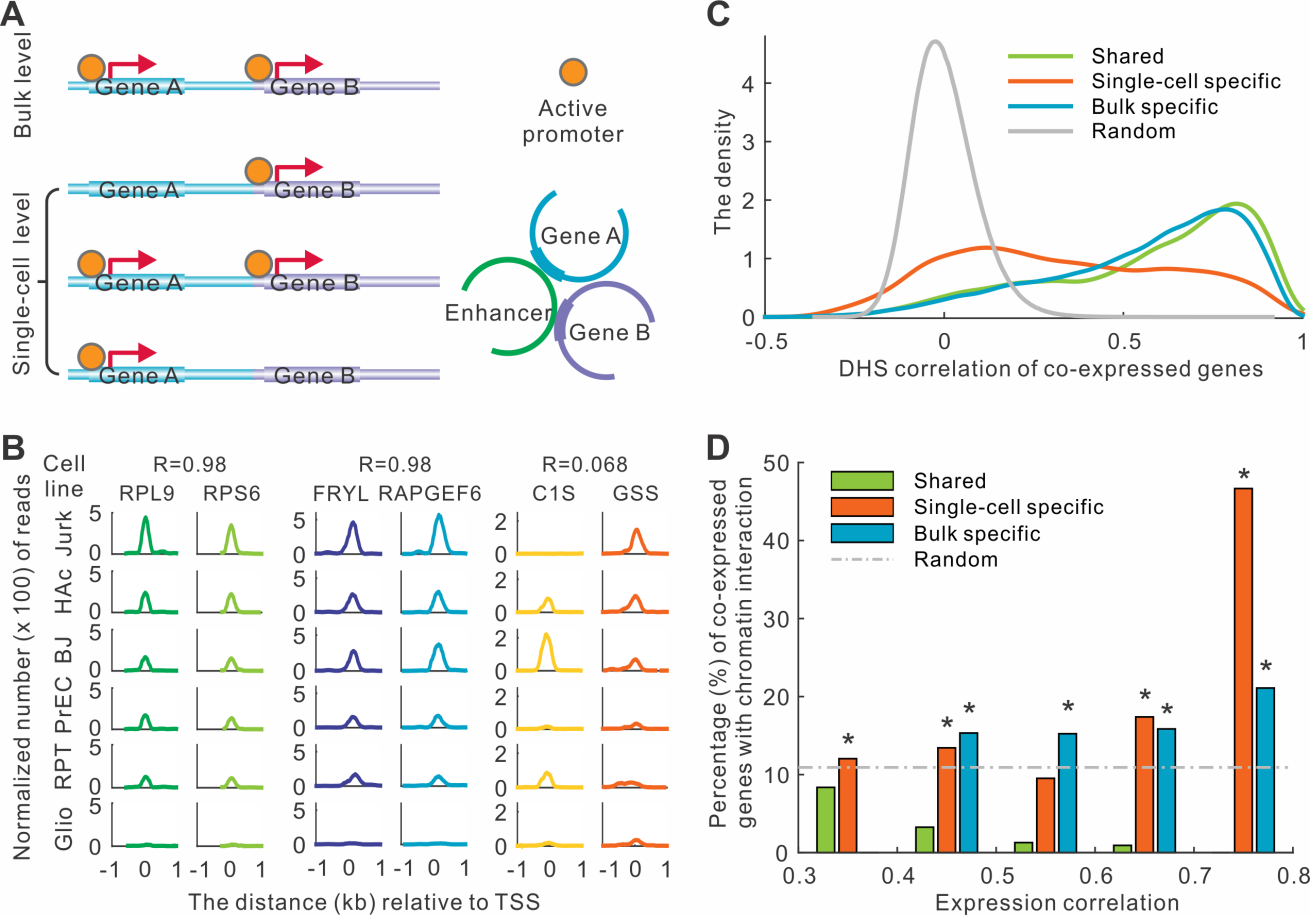
Distinct regulatory mechanisms are associated with co-expressions in single cells and bulk tissues. (A) Two models for co-regulation. Two genes which were detected to have synchronized promoters at the bulk level may not be simultaneously regulated at the single-cell level. Two genes interacting with each other in 3-D chromatin may be co-regulated by the same enhancer. (B) Examples of DHS correlations in three types of co-expressed gene pairs. The figure showed six cell lines as examples. The correlation coefficients (R) were calculated based on 125 cell lines. (C) The distribution of the correlation coefficients of DHS signals across 125 cell types between co-expressed genes. (D) Fraction of co-expressed genes that have genomic interactions.

To determine whether the underlying regulatory mechanism of co-expression at single-cell level is different from those at the bulk level, we analyzed the possible regulatory relationships for the three groups of co-expressed genes. We examined two distinct and complementary mechanisms for co-expression (Fig 4A). First, we tested whether the co-expressed genes tend to have synchronized activity of their *cis*-regulatory elements across different physiological conditions. Second, we checked whether the *cis*-regulatory elements governing each pair of co-expressed genes more likely have physical contact in the three-dimensional nuclear space.

We computed the accessibility of gene promoters annotated by DNase I hypersensitive sites (DHSs) and corresponding DHS signal correlations across 125 human cell types and tissues [23]. Our analysis revealed that shared and bulk specific co-expressed gene pairs had significantly higher DHS correlation than the random expectation. For example, *RPL9* and *RPS6* belong to the shared co-expressed gene group, and the accessibility of their promoters was perfectly synchronized across the 125 cell types (R = 0.98, Fig 4B). Similarly, the accessibility of another pair, *FRYL* and *RAPGEF6*, bulk specific co-expressed genes, was also highly correlated (R = 0.98). Overall, the highest peak of the distribution of DHS correlation for shared and bulk specific co-expressed gene pairs were located at 0.81 and 0.79, respectively (Fig 4C). In contrast, the correlation coefficient for single-cell specific co-expressed genes was much lower than the other two groups (P < 1.0E-300, student’s t-test). For example, DHS signal of two genes, *GIS* and *GSS*, was not correlated (R = 0.068, Fig 4B). The correlation coefficients of single-cell specific genes were much broader distributed, with the highest peak located at 0.12 (Fig 4C).

We then calculated the probability that two genes physically interact with each other based on chromatin interaction data [24]. In IMR90 cell lines, we discovered that the single-cell specific and bulk specific co-expressed genes were more likely to have physical interactions than expectation (Fig 4D). In contrast, the shared co-expressed genes were not enriched for chromatin interactions (Fig 4D). The same observation was confirmed in an independent cell line of hESC (S10 Fig). Our results demonstrated that the datasets obtained from bulk tissues (e.g. DHS and chromatin interactions) could partially explain the co-expression at bulk and single-cell levels, and different types of co-expressions might be regulated by different mechanisms.

### Most of single-cell co-expressed genes were from different chromosomes

**Fig 5:**
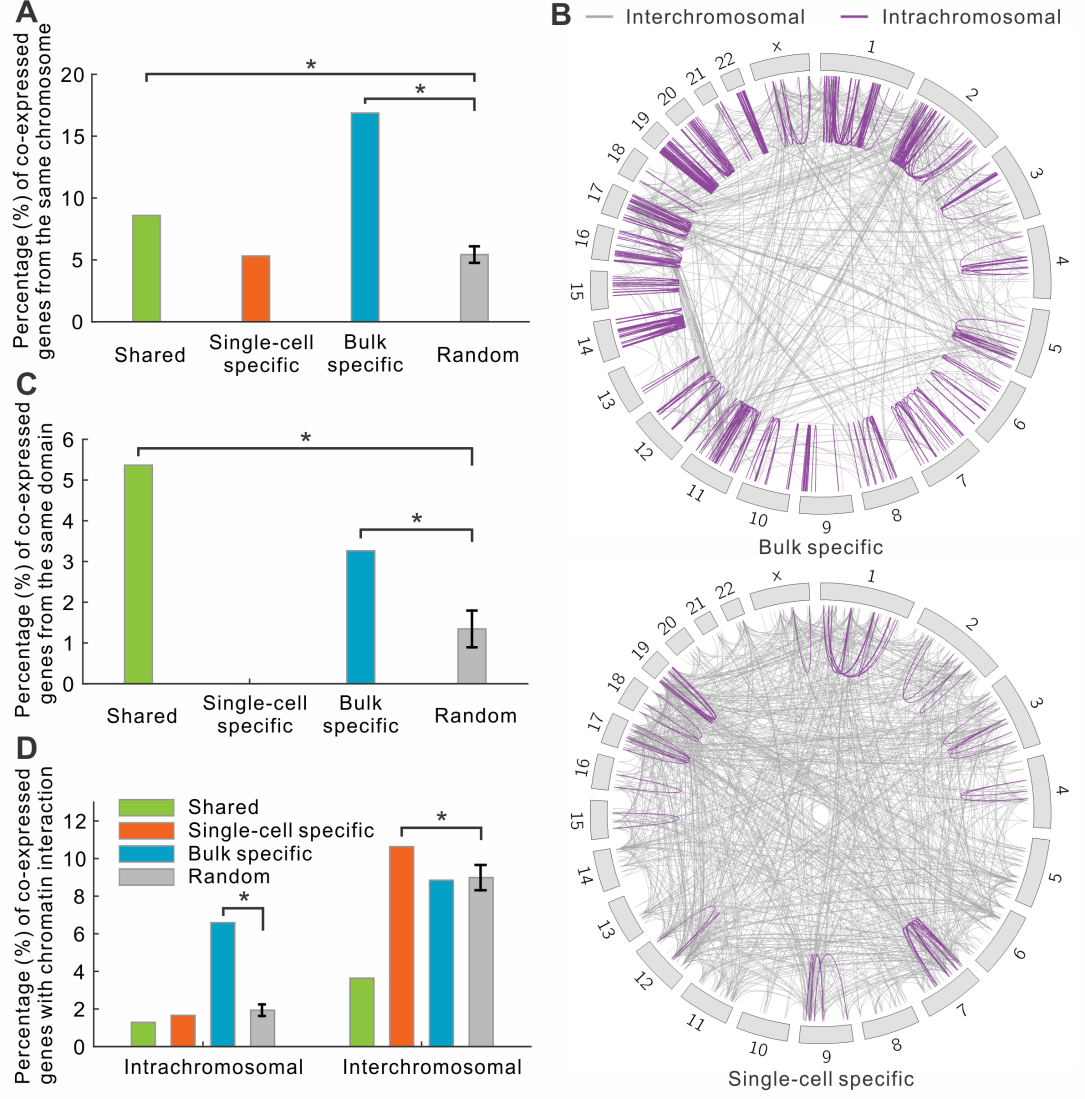
Interchromosomal interactions are prevalent in single-cell co-expressed genes. (A) The fraction of co-expressed gene pairs located in the same chromosomes. (B) Map of the co-expressed gene pairs. Each line represent a pair of co-expressed genes. The color of the lines indicates whether they are in the same chromosomes: purple (intrachromosomal), and gray (interchromosomal). (C) The fraction of co-expressed gene pairs located in the same topological domains. (D) The fraction of intrachromosomal and interchromosomal co-expression genes with chromosomal interactions.

Previous co-expression studies at the bulk level have shown that genes within the same topological domain were more likely to interact with each other [24]. Here we asked whether a pair of co-expressed genes resided on the same chromosome or even within the same topological domain. Interestingly, for bulk specific co-expressed genes, we observed that 16.9% were found on the same chromosome, whereas only 5.3% of single-cell specific co-expressed genes were encoded on the same chromosome, which was almost the same as randomly selected gene pairs (average 5.4%, Fig 5A). If we only focused on the top 1,000 highest co-expressed gene pairs, the difference between two levels became even more significant, 47.5% and 5.5% of bulk and single-cell specific genes were located in the same chromosome, respectively (Fig 5B). We further asked to what degree the intrachromosomal co-expressed genes were from the same topological domain [24]. Our analysis revealed that 3–6% of shared and bulk specific intrachromosomal co-expressed gene pairs were located at the same topological domain (Fig 5C). By contrast, no single-cell specific gene pairs were from the same topological domain.

When we separated the co-expressed genes based on whether they were encoded on the same chromosomes, we found that the interchromosomal chromatin interactions were enriched for single-cell specific co-expressed genes (Fig 5D). This result suggests that many co-expressed genes in single cells were co-regulated through interchromosomal interactions, by which the *cis*-regulatory elements of genes were physically connected and co-regulated by common regulators such as enhancers (Fig 4A).

### Co-expressed genes could serve as a prognosis signature for glioblastomas

**Fig 6:**
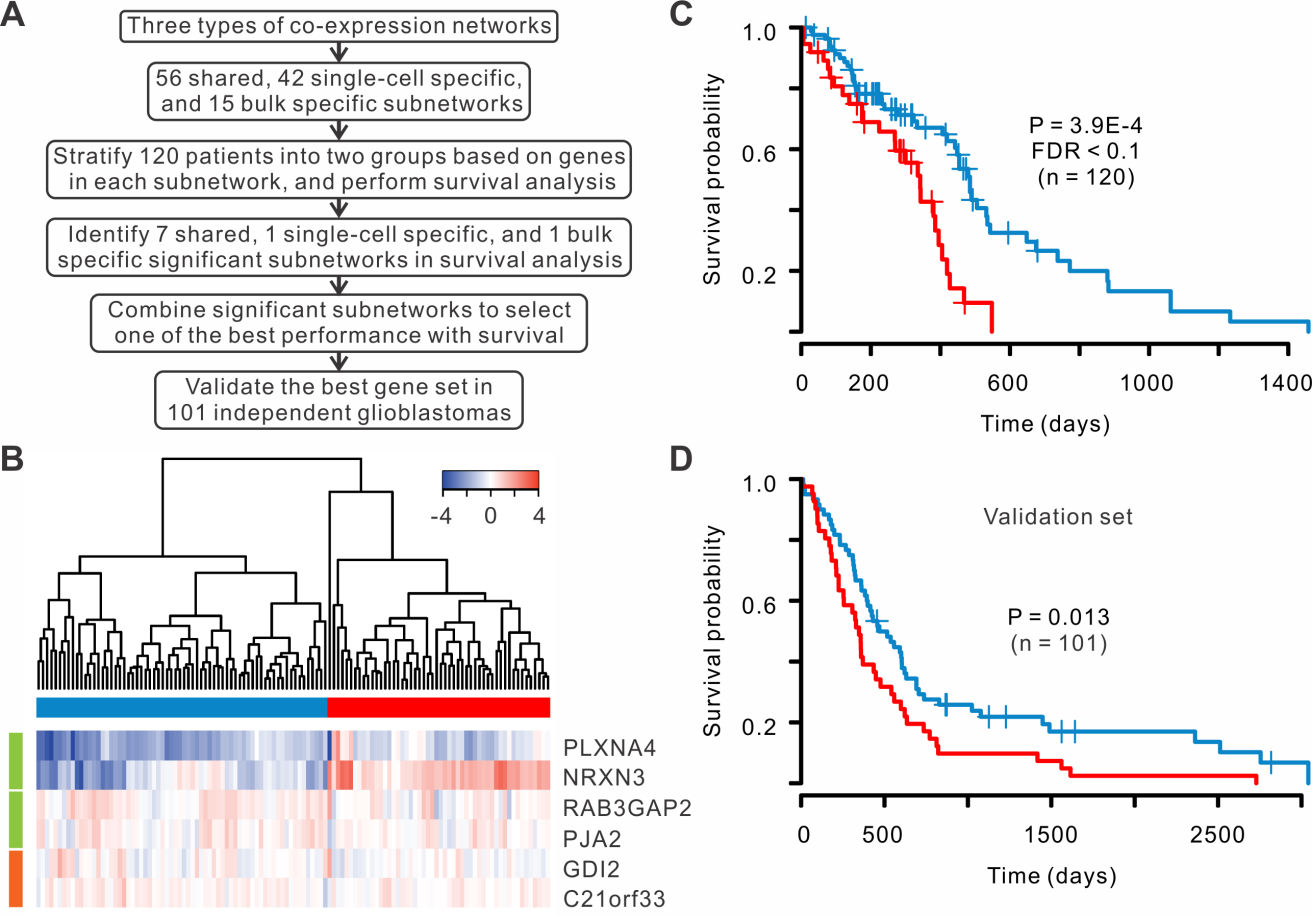
A set of co-expressed genes can serve as a prognosis signature for glioblastomas. (A) Flow chart of the selection of co-expressed genes for prognosis analysis. (B) Six genes were selected to classify the patients. Four genes are shared co-expressed (green), and two genes are single-cell specific co-expressed (orange). (C) Kaplan-Meier survival curves in two groups of 120 sequencing samples. Log-rank test was used. (D) Survival curves for an independent validation set.

Recent studies demonstrated that network-based classification approaches provided more power in prediction of clinical outcomes than individual genes [25–27]. We searched the subnetworks within the three types of co-expression networks to identify a set of co-expressed genes that could stratify patients with most significantly different survival time. We classified 120 patients with RNA sequencing from TCGA into two groups based on the expression profiles of genes within each subnetwork (or combination of subnetworks) and compared the survival rates between the two groups (Fig 6A). By examining all subnetworks and the combination of the subnetworks, we discovered a combination that achieved the best separation of patient survival rates, which consisted of 4 shared and 2 single-cell specific co-expressed genes (Fig 6B). The two groups of patients were well separated based on the silhouette plot (S11 Fig). The survival rates of the two groups were significantly different (P = 3.9E-4, log-rank test, FDR < 0.1, Fig 6C). As comparison, we performed the same analysis to bulk co-expressed genes, but no subnetwork was found to classify the patients with significant difference in survival rates (S12 Fig), suggesting that single-cell expression profiles help to improve the prognosis of glioblastoma. Furthermore, we classified the patients into four subtypes according to TCGA classification scheme [28], and their survival rates were not significantly different (S13 Fig).

To confirm the classification power of co-expressed genes, we tested our gene signature using an independent set of 101 glioblastomas whose expressions were profiled using microarray from TCGA. The validation indicated that six-gene signature could significantly stratify poor and favorable survival of the patients (P = 0.013, log-rank test, Fig 6D). These results suggest that the co-expressed gene signature has a great potential to predict patient survival.

## Discussion

Our analysis revealed distinct characteristics for the co-expressed genes at single-cell and bulk levels. The stark difference between the two levels suggests that the single-cell expression profiles provide novel biological insights when they are compared with bulk expression profiles. Interestingly, the DHS and chromatin interaction datasets obtained from bulk tissues could partially explain the co-expression at single-cell level. Nevertheless, we are fully aware of the difference of gene regulation between bulk and single-cell levels. For example, two bulk co-expressed genes could have the same accessibility of regulators in their promoters, whereas the regulation of the two genes at single-cell level is independent to each other and could result in un-correlated accessibilities of the promoters (Fig 4A). If we could deconvolute the signal from the bulk tissues or obtain the datasets on gene regulation at single-cell level, we expect to obtain stronger connection between co-expression and co-regulation. Although a few DHS or ChIP-seq datasets at single-cell level are available [29–31], the data quality is still not optimal (e.g. low sequencing depth). One interesting observation is that majority of the single-cell co-expressed genes are located in different chromosomes, in line with a recent observation that co-expressed odorant receptor genes was not restricted to single chromosome at single-cell level [32]. While the current chromatin interaction analyses are mainly focused on intrachromosomal interactions [33,34], our analysis suggests that interchromosomal interactions are of biological interests.

In our analysis, a set of six co-expressed genes was used to stratify glioblastoma patients into two groups with significantly different survival. Although these genes were selected without prior knowledge of cancer biology, the genes are relevant to glioblastomas. For example, *PLXNA4* (plexin A4) has been shown to promote tumor angiogenesis and progression of glioblastoma cells [35]. Similarly, *NRXN3* (Neurexin 3) was involved neuron cell-cell adhesion and glioma cell migration [36]. Gene *RAB3GAP2* (RAB3 GTPase activating non-catalytic protein subunit 2) was implicated in neurodevelopment and Warburg Micro syndrome [37], whereas *PJA2* (praja ring finger ubiquitin ligase 2) degraded MOB1 to support glioblastoma growth [38]. Moreover, both *GDI2* (GDP dissociation inhibitor 2) and *C21orf33* (chromosome 21 open reading frame 33) was dysregulated in fetal Down syndrome brain [39,40]. All the genes were related to glioblastoma or neural diseases, suggesting their underlying function in tumorigenesis and progression of glioblastoma.

## Materials and Methods

### Expression profiles of single cells and bulk tissues

Single cell expression datasets were obtained from references [14,19]. For glioblastoma, 430 individual cells from 5 patients were sequenced for gene expression. For prostate cancer, 122 cells from 22 patients were sequenced. Bulk expression datasets were obtained from The Cancer Genome Atlas (TCGA, https://tcga-data.nci.nih.gov/tcga/). In total, 120 glioblastomas and 398 prostate adenocarcinomas were measured by RNA sequencing at the bulk level.

For bulk expression profile, we excluded the genes whose average expression was below 100 RPKM (Reads Per Kilobase per Million mapped reads). For single-cell gene expression, we excluded the genes if the expression levels across over two-thirds individual cells were equal to zero. Only the genes that were measured at both single-cell and bulk levels were included for further analysis. In total, 4,837 and 4,722 genes were analyzed for glioblastoma and prostate adenocarcinomas, respectively. We performed log2-transformation for RPKM. In order to avoid 0 value for invalid log2-transformation, we added 1 to RPKM value. We then performed global centralization by subtracting corresponding average expression across tissues or cells. Quantile normalization of the expression was further conducted across all samples. All analyses were performed in R platform (http://www.r-project.org/).

### Co-expression analysis and hierarchical clustering

Pairwise correlations for all genes were calculated using Pearson correlation coefficient (R). The formula is as follows

$$R=\frac{\sum_{i=1}^{n}{(x}_{i}-\bar{x})(y_{i}-\bar{y})}{\sqrt{\sum_{i=1}^{n}{{(x}_{i}-\bar{x})}^{2}}\sqrt{\sum_{i=1}^{n}{{(y}_{i}-\bar{y})}^{2}}}$$

where *x*, *y* are gene pairs, and *n* is the sample size. All gene pairs were ranked according to R values. The hierarchical clustering of expression profiles took Pearson’s correlation coefficient as similarity measurement, and used complete linkage. Similarly, we also used MIC (maximal information coefficient) to measure expression correlation of gene pairs [18].

We then classified the co-expressed genes into three groups: bulk specific, single-cell specific, and shared. Since the distributions of correlation coefficients are quite different between single cells and bulk tissues, we could not choose a uniform cutoff to define the positive correlation. Instead, we developed a shuffled-expression-based algorithm to determine the cutoffs for single-cell and bulk expression separately. Firstly, we shuffled the expression for each gene across the samples, and generated a corresponding distribution of correlation coefficients. We then set the correlation coefficient at the top percentage of 10^−6^ as cutoff for positive correlation. After setting the cumulative probability of no correlation in random distribution to 0.3 for each side around zero correlation, we obtained the cutoffs of no correlation. The criteria for positive correlation and no correlation are very stringent here because we want to make sure the selected groups of gene pairs are indeed bulk specific or single-cell specific. Those positively correlated gene pairs at both single-cell and bulk levels were assigned to the group of shared co-expressed genes. Single-cell specific co-expressions were those gene pairs with positive correlation at the single-cell level but no correlation at the bulk level. Similarly, those gene pairs with positive correlation at the bulk level but with no correlation at the single-cell level were assigned to bulk specific co-expressed genes.

### Integration with protein-protein interactions and protein complexes

In order to associate gene co-expressions with protein-protein interactions (PPIs), we downloaded PPIs from BioGrid [20]. We calculated the fraction of co-expressions with PPIs in each type of co-expressed genes. Meanwhile, we generated one thousand sets of 1,000 pairs of genes randomly selected from all gene pairs as control gene pair sets. Each set of control gene pairs were associated with PPIs as well. To calculate the proportion for different ranges of expression correlations, we divided co-expressed genes into equal-interval groups with 0.1 bin size of the correlations.

The components of protein complexes were from CORUM database [41]. All shared and specific co-expressions were mapped to each protein complexes. The layout and view of co-expression network of protein complexes were performed in Cytoscape [42].

### Analysis of GO similarity and function enrichment

We used R package GOSemSim to calculate the semantic similarity of the biological process (BP) terms of gene ontology (GO) [22] between two genes. If similarity value of gene pair ≥ 0.5, the genes were called with GO similarity. Based on this criterion, we calculated the percentage of co-expressed gene pairs and randomly selected gene pairs with GO similarity.

To identify the enriched GO terms, we each chose the top 1,000 co-expressed gene pairs from three groups of co-expressions, respectively. We obtained 129 unique genes from top 1,000 shared co-expressions. After excluding the genes were overlapped between shared and single-cell specific co-expressions, we obtained 319 single-cell specific genes. Besides, 640 genes were unique to bulk specific co-expressions. These three groups of genes were separately performed function enrichment analysis through DAVID software [43]. According to the enriched functions, co-expression networks of top 1,000 correlations were organized into different modules. The genes were assigned to the most significant module if they were enriched in multiple functional modules.

### DHS correlation, chromatin interactions and location of co-expressed genes

DNase I hypersensitive sites (DHSs) in 125 human cells and tissues were downloaded from ENCODE project [23]. DHSs within the promoter regions (upstream 1,000 base pairs relative to transcription start sites (TSSs)) were associated to genes. If no DHS peaks were found within the promoter regions, the intensity of DHSs of genes was assigned to zero. We then calculated DHS correlations of gene pairs across 125 cell types.

To identify chromatin interaction of co-expressed genes, we used Hi-C data from previous publication [24]. The DNA regions across upstream 5,000, gene body, and downstream 5,000 were used to identify whether gene pairs have chromatin interaction.

Chromosomal relationships of co-expressed gene pairs were plotted using Circus [44]. Topological domains in genome were reported by a previous study [24]. According to the locations of TSSs, gene pairs were determined whether they were located in the same chromosome or topological domain.

### Identification of gene signature for glioblastoma survival

We constructed three networks separately from single-cell specific, bulk specific and shared co-expressions. Using ‘Fast Modularity’ software [45], we then determined 56, 42, and 15 dense subnetworks within these three co-expression networks, which reflect the functionally related gene groups. For each subnetwork, we performed hierarchical clustering of patients based on the bulk expression levels of the genes within the subnetwork. The patients were classified into two groups according to the clustering and then compared of their survival using the Kaplan-Meier method [46]. The significance of differential survival between two groups of patients was assessed with a log-rank test. After testing all the subnetworks, 7 shared, 1 single-cell specific and 1 bulk specific subnetworks were found to be able to separate the patients with significantly different survival rates (P < 0.05, log-rank test). We then further examined the combination of at most three significant subnetworks using the same procedure and discovered one combination with the best performance for tumor prognosis. We then estimated the false discovery rate (FDR) using Benjamini and Hochberg approach [47]. The quality of partition of patients was assessed through silhouette graph [48].

In TCGA, another 123 glioblastomas were measured using microarray platform, of which 22 samples were also profiled with RNA sequencing. In order to make the expression levels comparable between microarray samples and sequencing samples, we used one of patients (TCGA-06-0156), which was measured both by RNA sequencing and microarray, for normalization. After log2-transformation of sequencing data, expression profile of each patient subtracted the average expression of TCGA-06-0156 from RNA sequencing. Similarly, expression profiles measured by microarray also subtracted the average expression of microarray-measured TCGA-06-0156. Using the gene signature obtained from the 120 glioblastomas, we then predicted the class of additional 101 microarray-measured glioblastomas (a validation set) through a nearest shrunken centroid [49].

## Supporting Information

S1 Fig Expression correlation of ATP9B and MORC4 at single-cell and bulk levels. Gene correlation at the single-cell level is separately showed for five glioblastomas. Pearson’s correlation coefficient (R) and corresponding P value are indicated in the panel.
(TIF)

S2 Fig Expression correlation of REST and ROCK2 at single-cell and bulk levels. Gene correlation at the single-cell level is separately showed for five glioblastomas. Pearson’s correlation coefficient (R) and corresponding P value are indicated in the panel.
(TIF)

S3 Fig Expression correlation of RPL41 and RPS14 at single-cell and bulk levels. Gene correlation at the single-cell level is separately showed for five glioblastomas. Pearson’s correlation coefficient (R) and corresponding P value are indicated in the panel.
(TIF)

S4 Fig The distribution of top maximal information coefficients at single-cell and bulk levels of glioblastomas. Green, orange, and cyan lines represent shared, single-cell specific, and bulk specific correlations, respectively.
(TIF)

S5 Fig Scatter plot of average and bulk-level expression in glioblastoma. Each point represents a gene.
(TIF)

S6 Fig The distribution of gene correlations at single-cell and bulk levels in prostate cancer. Green, orange, and cyan lines represent shared, single-cell specific, and bulk specific co-expressions, respectively.
(TIF)

S7 Fig The division of correlation patterns at single-cell and bulk levels. The cutoffs of negative, no and positive correlations (vertical dashed lines) were set according to 1,000 times of the distributions of gene correlations of shuffled expression (only one example showed: R-S-MGH and R-B-GBM for single-cell and bulk levels, respectively). The shared, single-cell specific, and bulk specific co-expressions are highlighted in dash-dotted lines.
(TIF)

S8 Fig The number of gene pairs in each correlation pattern. The symbols ‘+’, ‘0’, and ‘-’ separately represent positive, no, and negative correlation. The three groups of gene pairs which are shared, single-cell specific, and bulk specific co-expressions are highlighted in green, orange, and cyan color, respectively.
(TIF)

S9 Fig Enriched functions of three types of co-expressed genes. The significant value for term ‘Translation elongation’ is equal to 90 and truncated for view. The bar-plot is corresponding to Fig 3C.
(TIF)

S10 Fig Chromatin interaction of co-expressed genes in hESC. The dash horizontal line represents an average percentage of control gene pairs with chromatin interaction. The asterisk indicates the percentage is significantly higher than control in statistics.
(TIF)

S11 Fig Silhouette plot of the division of patients. The figure shows that the patients are similar to other patients within the group than patients in another group. Each line represents a patients. The color of the line indicate the group of patients.
(TIFF)

S12 Fig Kaplan-Meier survival curves of 120 glioblastoma patients based on the best subnetwork from bulk co-expressed networks. The two-gene set was one of 13 subnetworks in bulk co-expressed network which divides 120 glioblastomas to two size-balanced groups. Log-rank test was performed to assess the significance of survival difference.
(TIF)

S13 Fig Kaplan-Meier survival curves of 120 glioblastoma patients based on four TCGA subtypes. Log-rank test was performed to assess the significance of survival difference.
(TIF)
