# Supplementary figures and images for: Single-Cell Co-expression Analysis Reveals Distinct Functional Modules, Co-regulation Mechanisms and Clinical Outcomes
Source: PLoS Comput Biol. 2016 Apr 21;12(4):e1004892. doi: 10.1371/journal.pcbi.1004892 (PMC4839722; doi:10.1371/journal.pcbi.1004892)

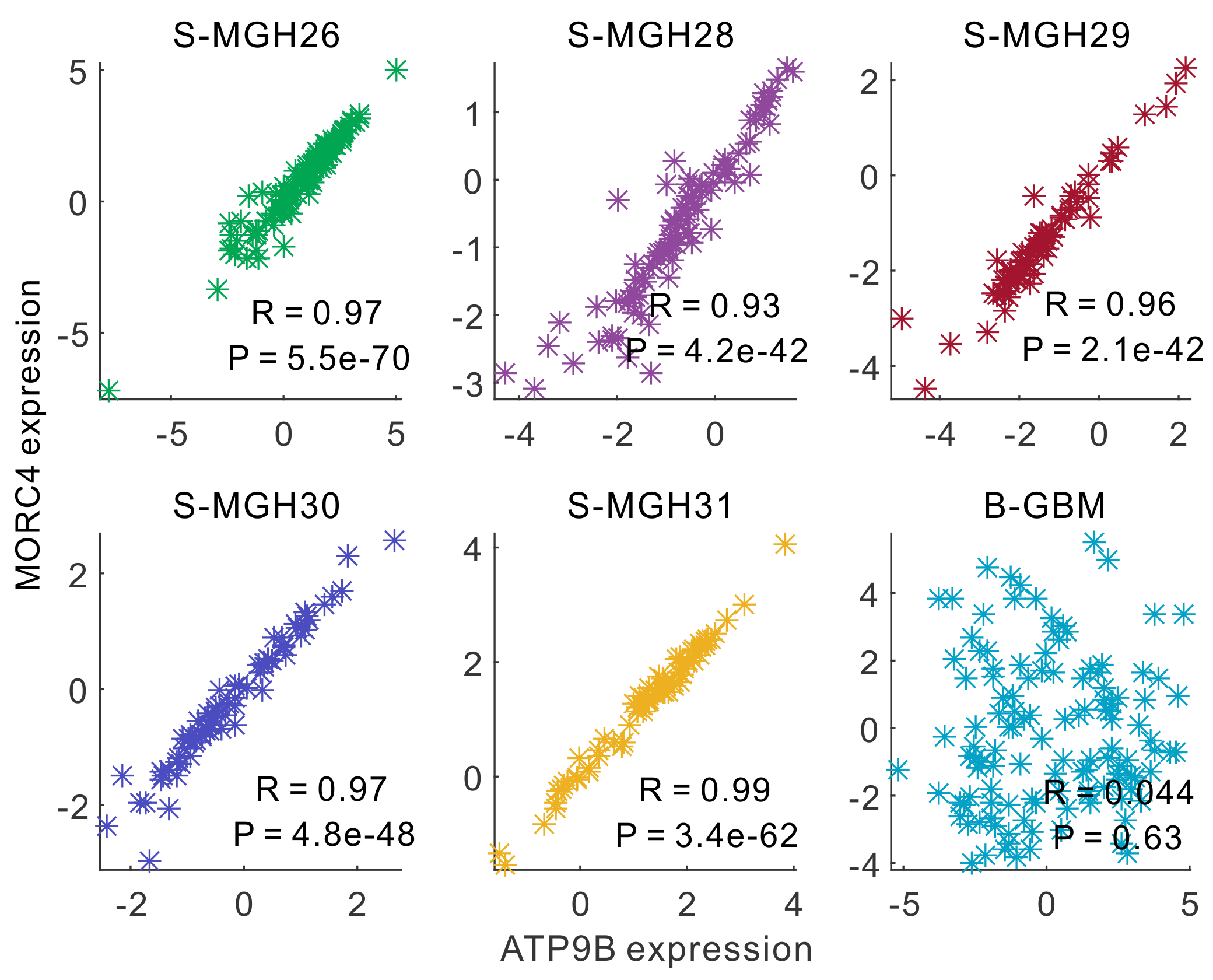

Supplement: S1 Fig — Gene correlation at the single-cell level is separately showed for five glioblastomas. Pearson’s correlation coefficient (R) and corresponding P value are indicated in the panel. (TIF) [file pcbi.1004892.s001.tif]

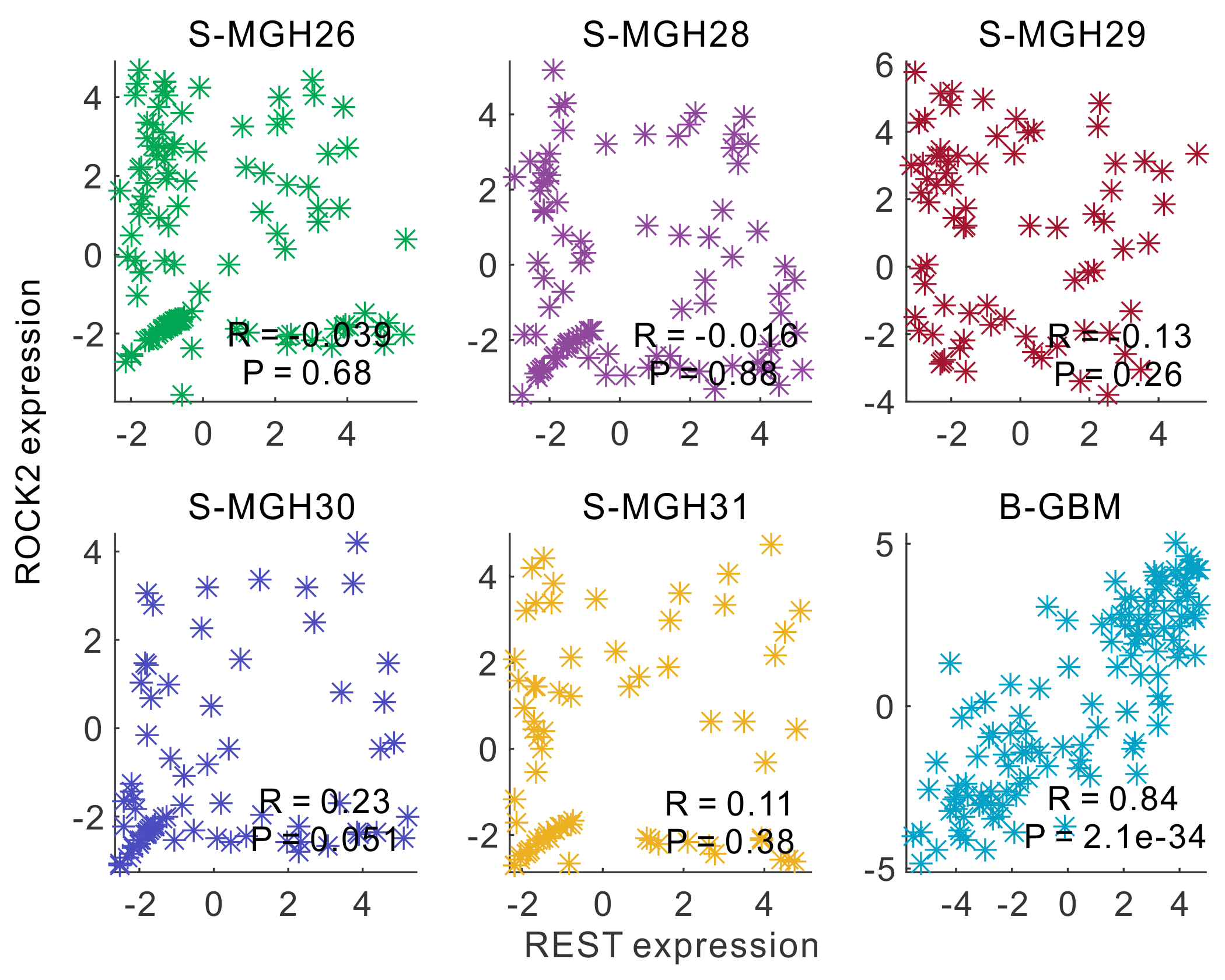

Supplement: S2 Fig — Gene correlation at the single-cell level is separately showed for five glioblastomas. Pearson’s correlation coefficient (R) and corresponding P value are indicated in the panel. (TIF) [file pcbi.1004892.s002.tif]

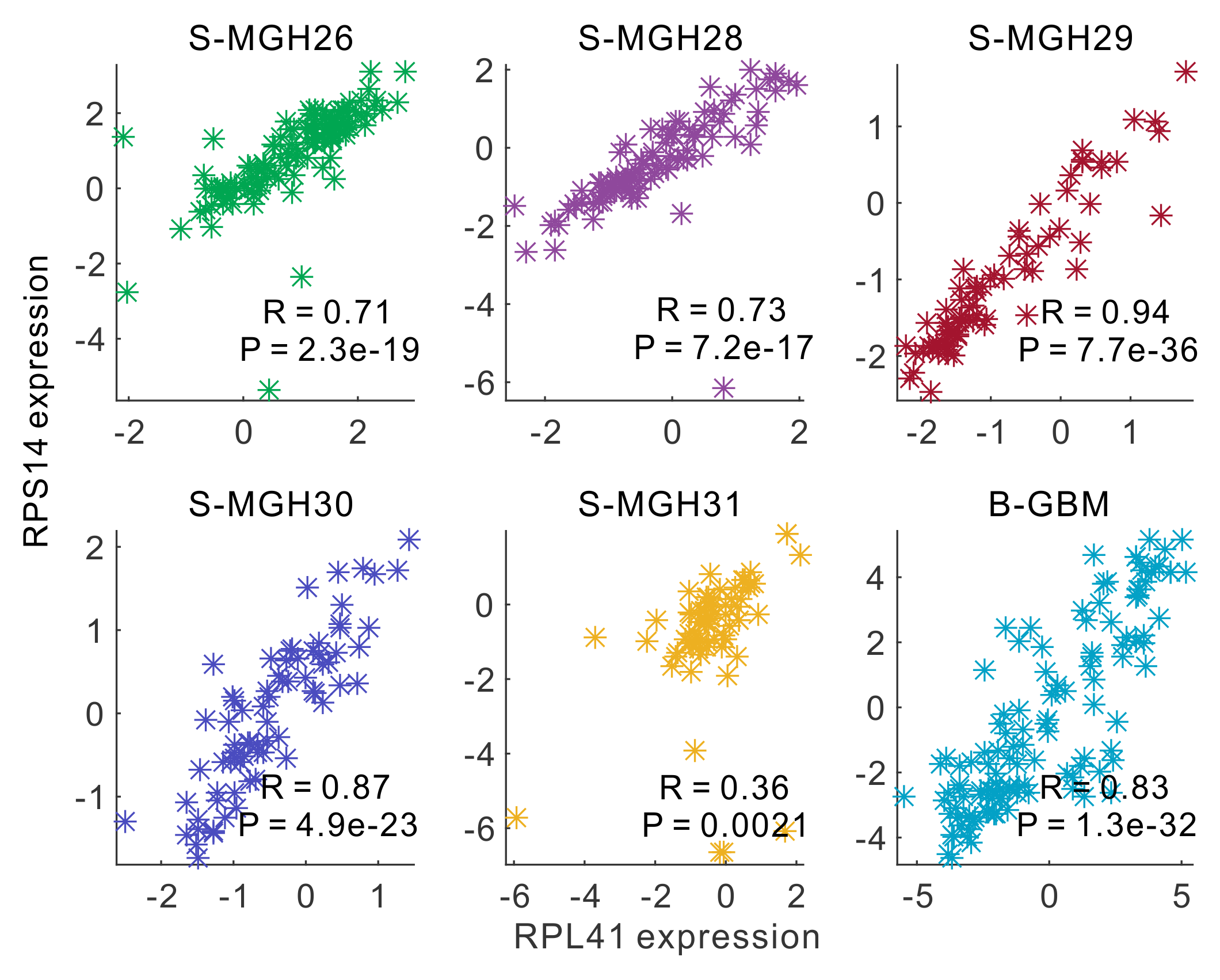

Supplement: S3 Fig — Gene correlation at the single-cell level is separately showed for five glioblastomas. Pearson’s correlation coefficient (R) and corresponding P value are indicated in the panel. (TIF) [file pcbi.1004892.s003.tif]

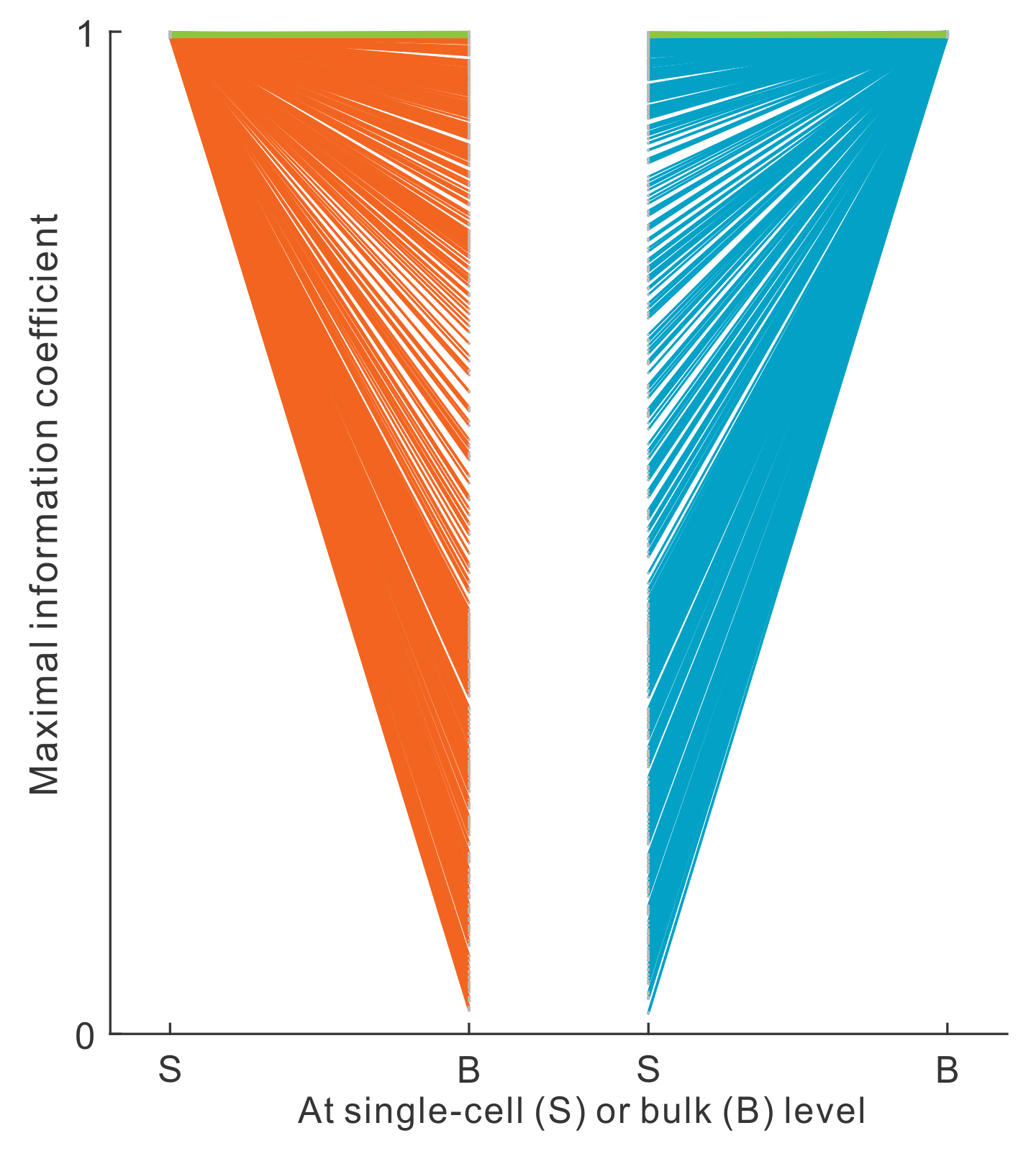

Supplement: S4 Fig — Green, orange, and cyan lines represent shared, single-cell specific, and bulk specific correlations, respectively. (TIF) [file pcbi.1004892.s004.tif]

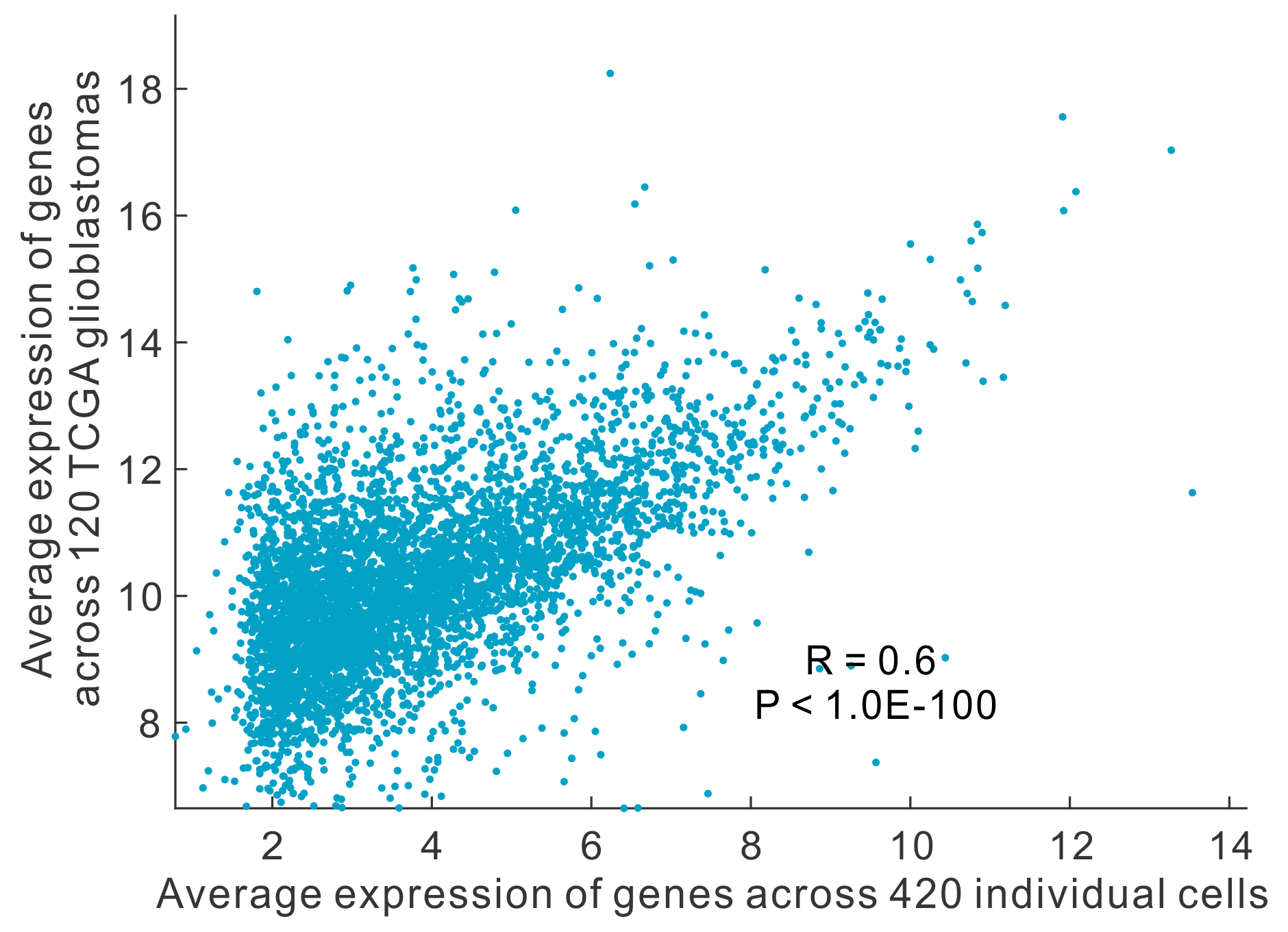

Supplement: S5 Fig — Each point represents a gene. (TIF) [file pcbi.1004892.s005.tif]

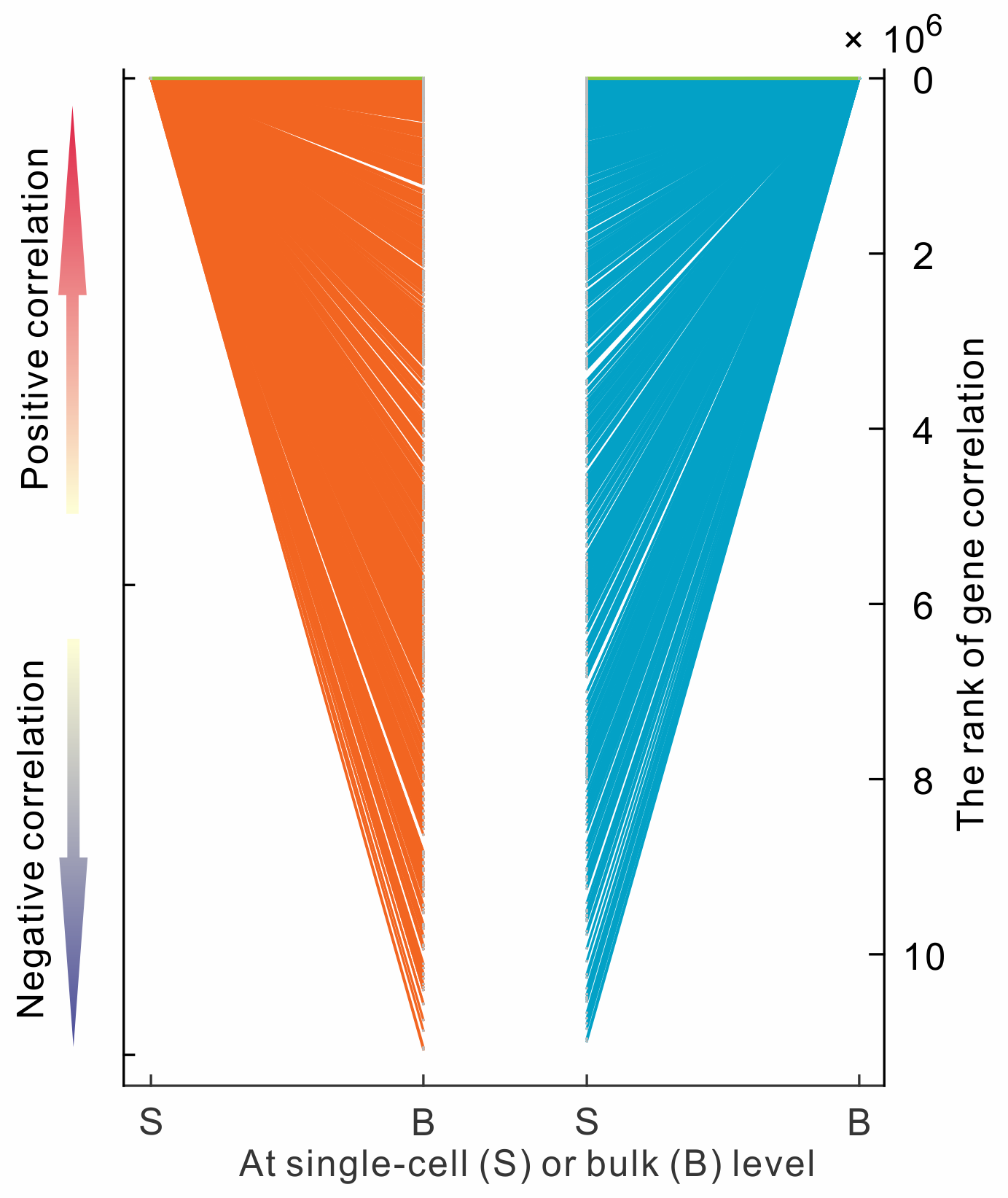

Supplement: S6 Fig — Green, orange, and cyan lines represent shared, single-cell specific, and bulk specific co-expressions, respectively. (TIF) [file pcbi.1004892.s006.tif]

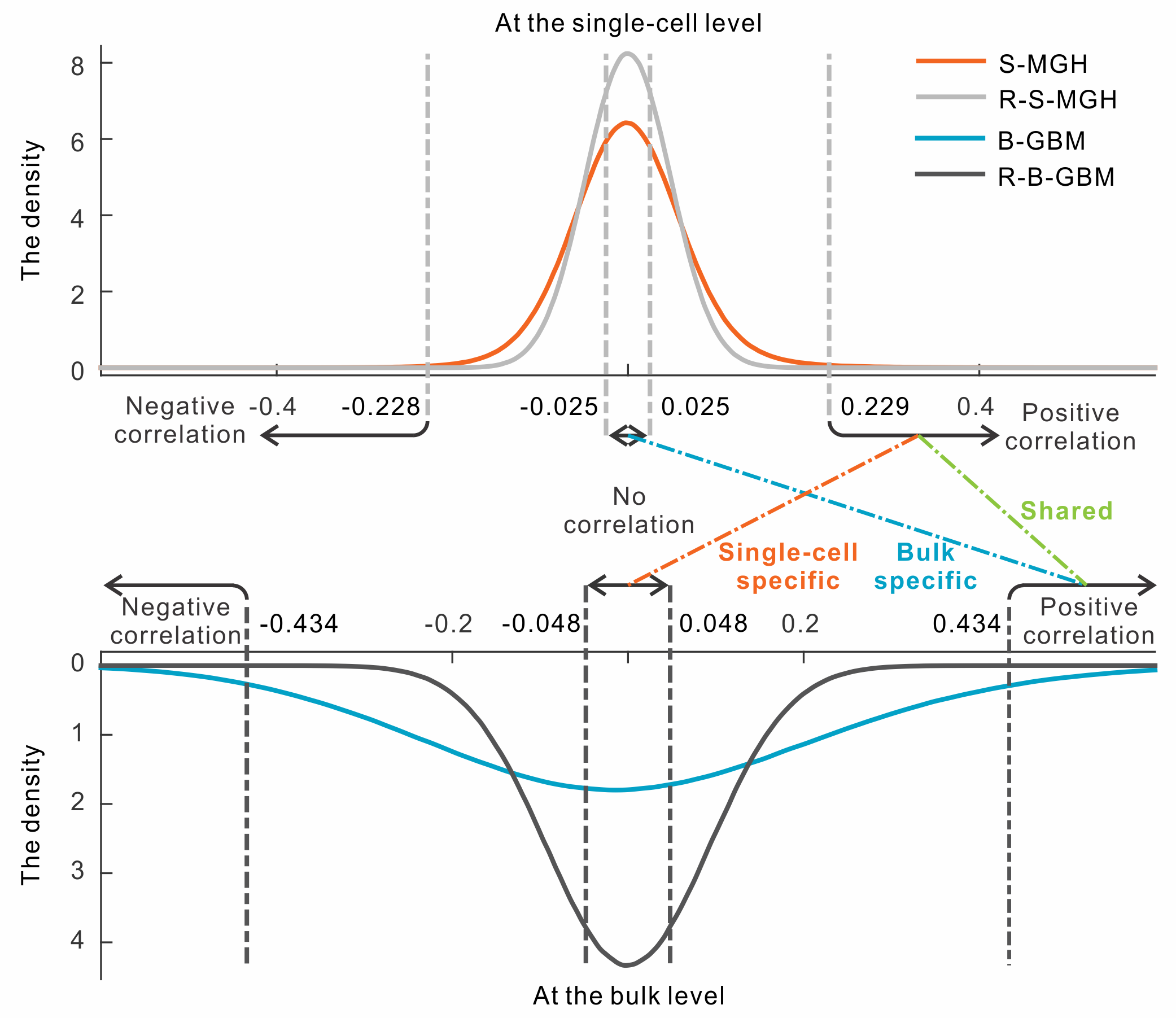

Supplement: S7 Fig — The cutoffs of negative, no and positive correlations (vertical dashed lines) were set according to 1,000 times of the distributions of gene correlations of shuffled expression (only one example showed: R-S-MGH and R-B-GBM for single-cell and bulk levels, respectively). The shared, single-cell specific, and bulk specific co-expressions are highlighted in dash-dotted lines. (TIF) [file pcbi.1004892.s007.tif]

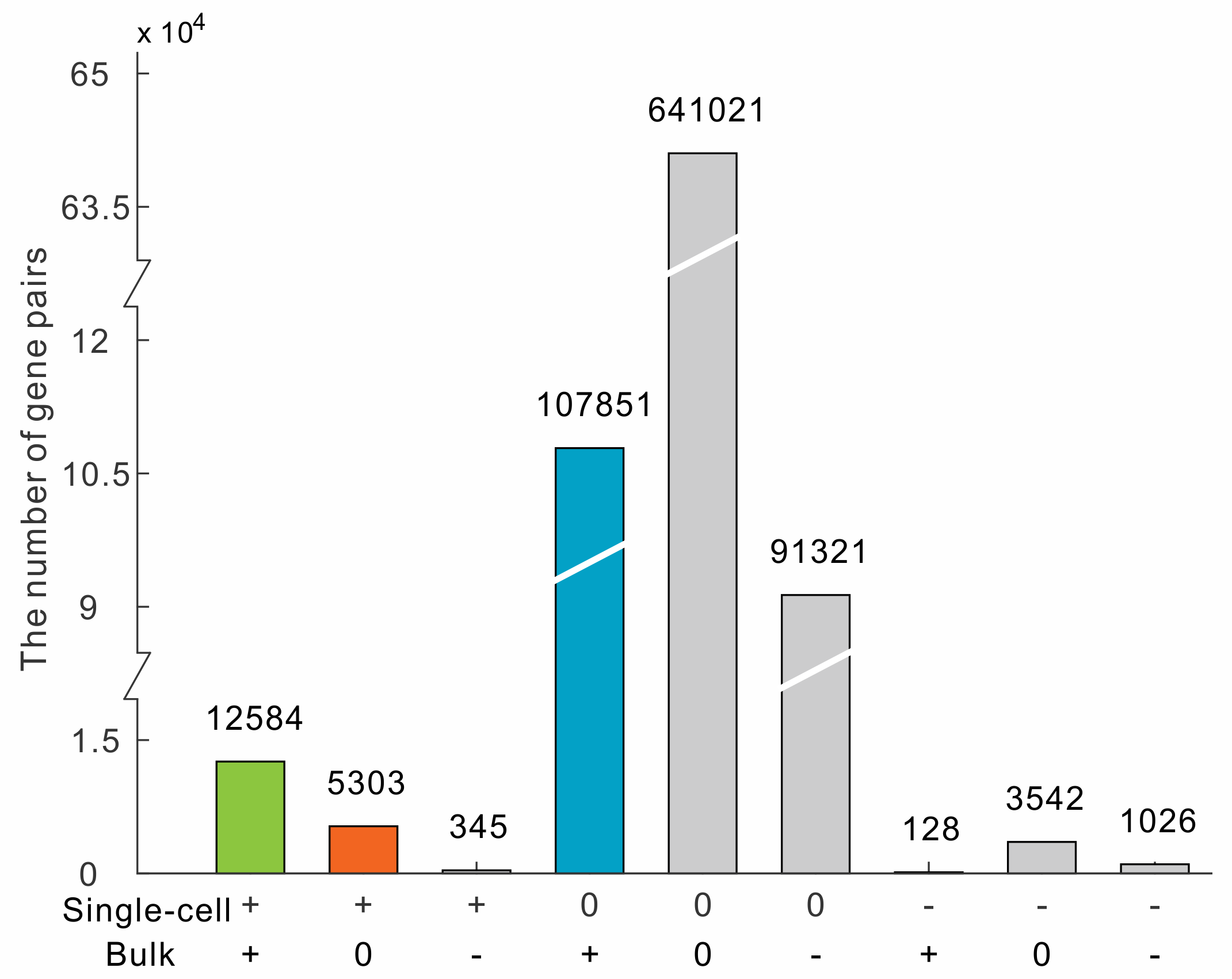

Supplement: S8 Fig — The symbols ‘+’, ‘0’, and ‘-’ separately represent positive, no, and negative correlation. The three groups of gene pairs which are shared, single-cell specific, and bulk specific co-expressions are highlighted in green, orange, and cyan color, respectively. (TIF) [file pcbi.1004892.s008.tif]

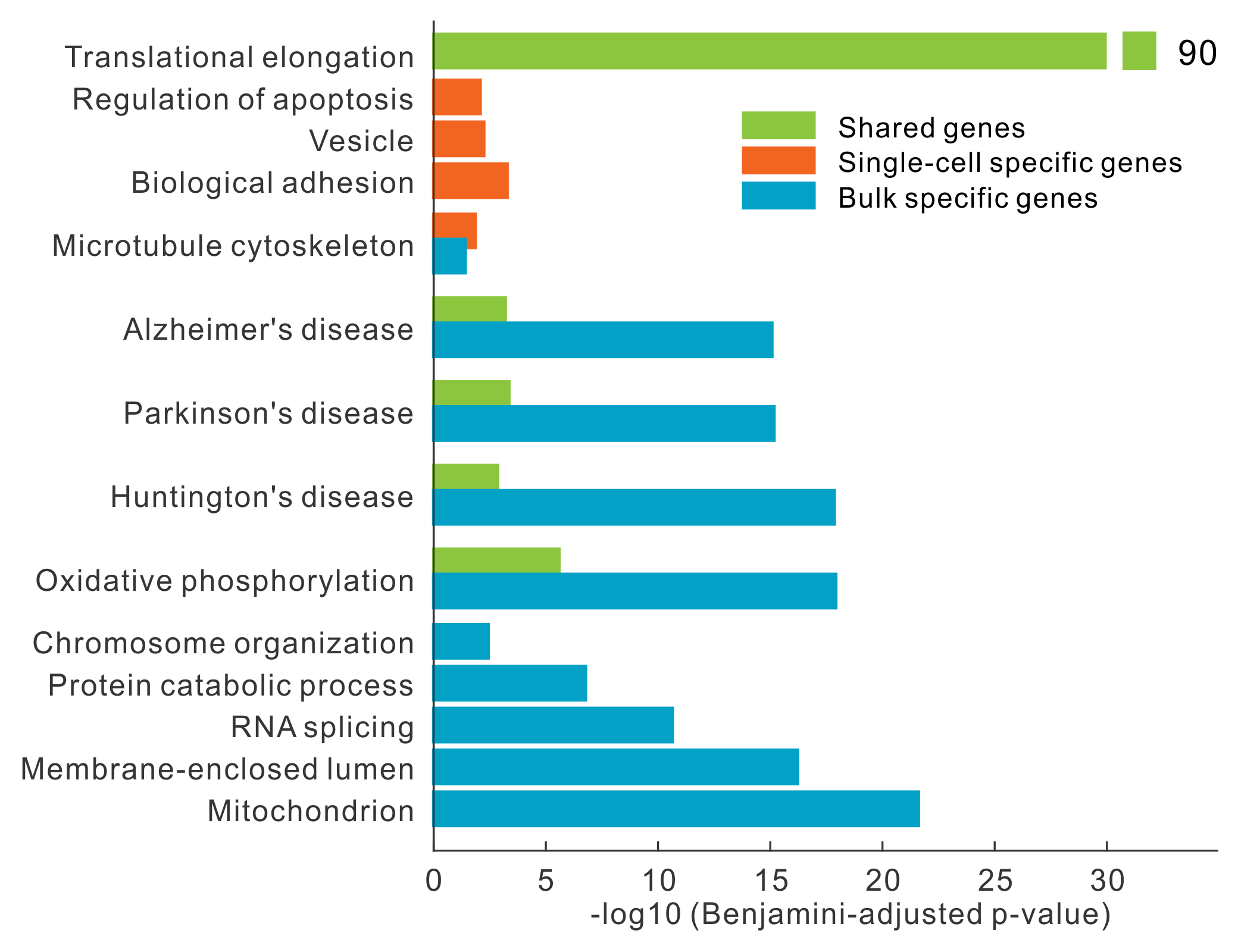

Supplement: S9 Fig — The significant value for term ‘Translation elongation’ is equal to 90 and truncated for view. The bar-plot is corresponding to Fig 3C. (TIF) [file pcbi.1004892.s009.tif]

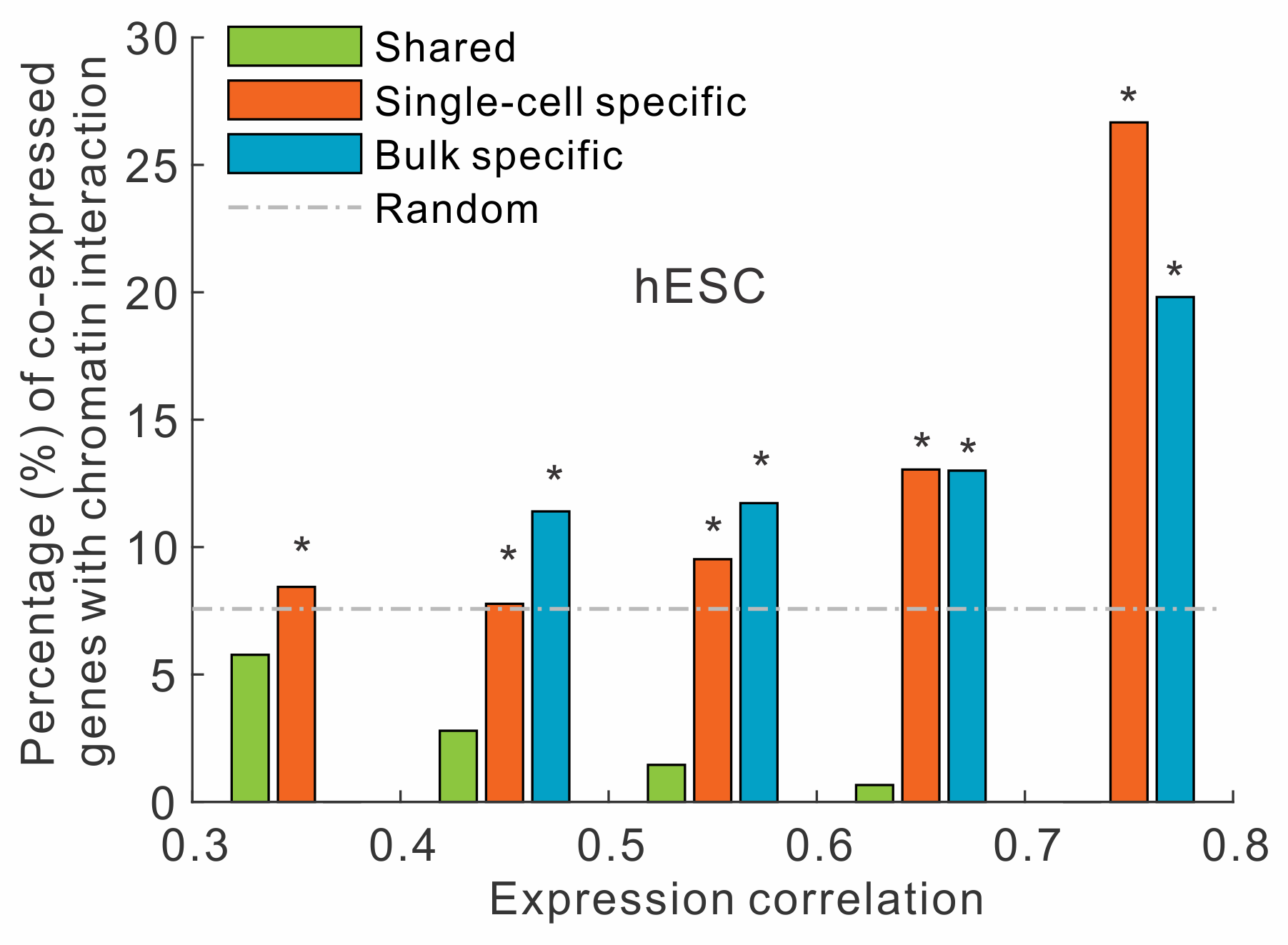

Supplement: S10 Fig — The dash horizontal line represents an average percentage of control gene pairs with chromatin interaction. The asterisk indicates the percentage is significantly higher than control in statistics. (TIF) [file pcbi.1004892.s010.tif]

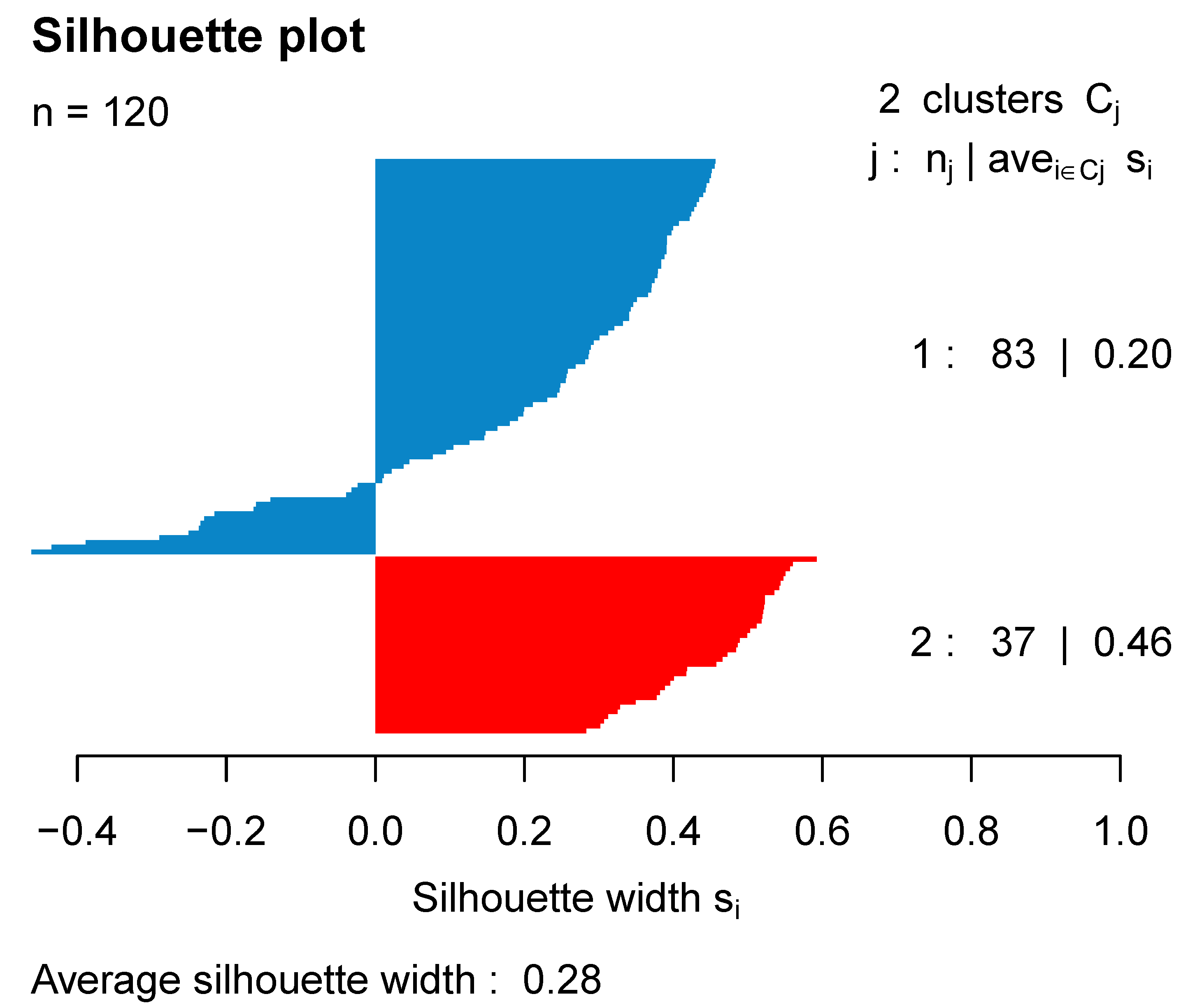

Supplement: S11 Fig — The figure shows that the patients are similar to other patients within the group than patients in another group. Each line represents a patients. The color of the line indicate the group of patients. (TIFF) [file pcbi.1004892.s011.tiff]

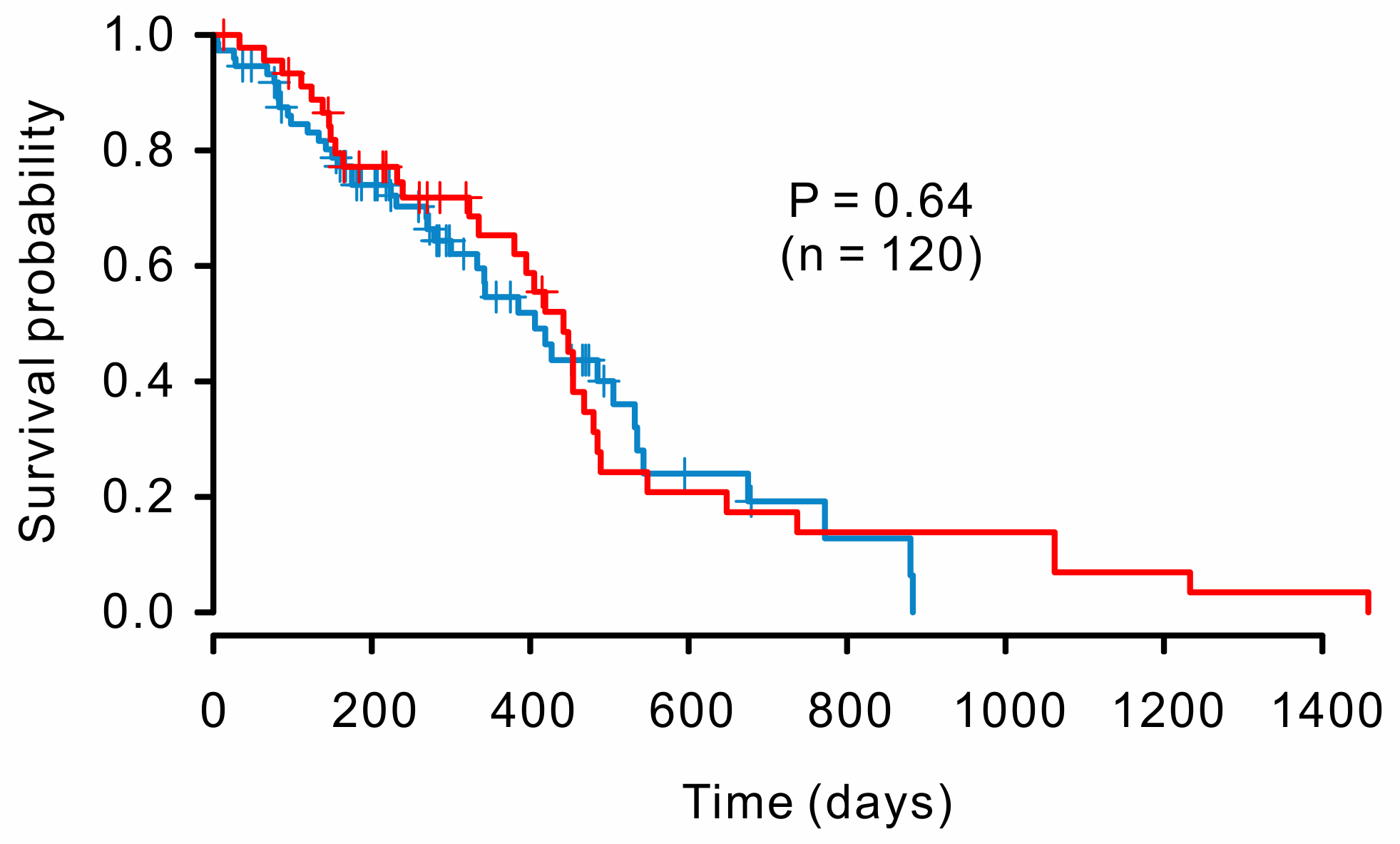

Supplement: S12 Fig — The two-gene set was one of 13 subnetworks in bulk co-expressed network which divides 120 glioblastomas to two size-balanced groups. Log-rank test was performed to assess the significance of survival difference. (TIF) [file pcbi.1004892.s012.tif]

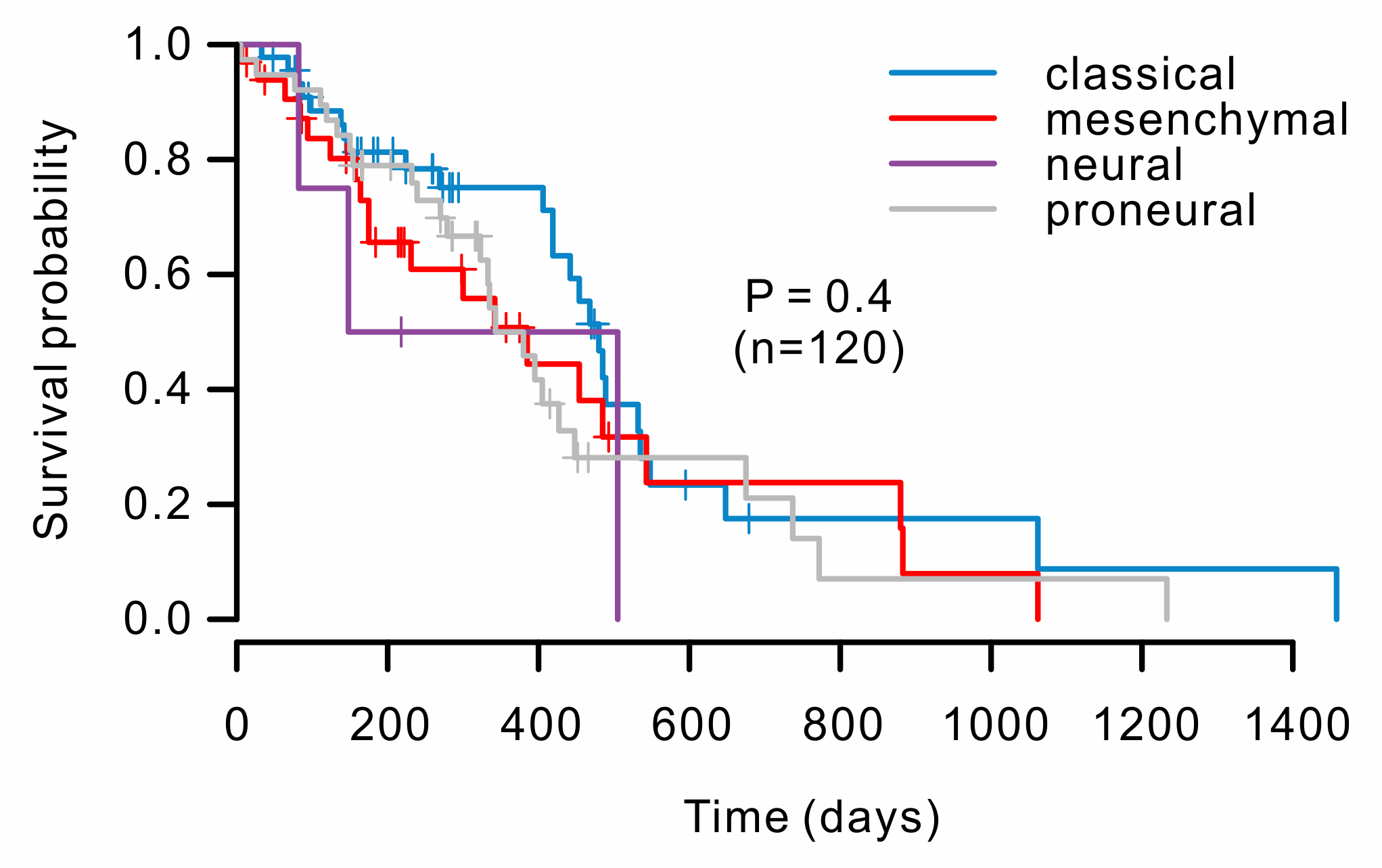

Supplement: S13 Fig — Log-rank test was performed to assess the significance of survival difference. (TIF) [file pcbi.1004892.s013.tif]
